# Effect of sodium-glucose cotransporter protein-2 inhibitors on left ventricular hypertrophy in patients with type 2 diabetes: A systematic review and meta-analysis

**DOI:** 10.3389/fendo.2022.1088820

**Published:** 2023-01-09

**Authors:** Yao Wang, Yujie Zhong, Zhehao Zhang, Shuhao Yang, Qianying Zhang, Bingyang Chu, Xulin Hu

**Affiliations:** ^1^ Clinical Medical College & Affiliated Hospital of Chengdu University, Chengdu University, Chengdu, Sichuan, China; ^2^ State Key Laboratory of Biotherapy and Cancer Center, West China Hospital, Sichuan University, Chengdu, Sichuan, China

**Keywords:** SGLT-2i, ventricular hypertrophy, type 2 diabetes, effectiveness, meta-analysis

## Abstract

**Objective:**

This systematic review and meta-analysis was performed to compare the effect of sodium-glucose cotransporter protein-2 inhibitors (SGLT-2i) and placebo on left ventricular hypertrophy (LVH) in patients with type 2 diabetes.

**Method:**

Randomized controlled trials (RCTs) comparing the LVH parameters of SGLT-2i to placebo in patients with type 2 diabetes were included. Our primary outcomes were the changes in left ventricular mass (LVM) and left ventricular mass index (LVMI) from baseline to the study endpoint. Secondary outcomes were the changes in left ventricular end-diastolic volume (LVEDV), left ventricular end-systolic volume (LVESV), left ventricular ejection fraction (LVEF), and the ratio of early mitral inflow velocity to atrial inflow velocity (E/A). Summary odds ratios were estimated using a fixed-effect or random-effect model.

**Results:**

A total of 11 articles were included. Data were extracted from 11 original studies matching our inclusion criteria. In our meta-analysis, there were significant improvement in LVM (SMD −0.23, 95% CI −0.44 to −0.02, *I* ^2^ = 22.6%, *p* = 0.034), LVMI (SMD −0.25, 95% CI −0.38 to −0.12, *I* ^2^ = 0.0%, *p* = 0.000), LVEDV (SMD −0.19, 95% CI −0.36 to −0.01, *I* ^2^ = 62.3%, *p* = 0.035), and LVESV (SMD −0.21, 95% CI −0.39 to −0.04, *I* ^2^ = 32.9%, *p* = 0.017) in the SGLT-2i group compared with the placebo group. Furthermore, no significant differences were found in LVEF (SMD 0.13, 95% CI 0.00 to 0.26, *I* ^2^ = 0.0%, *p* = 0.050) and E/A (SMD −0.01, 95% CI −0.22 to 0.20, *I* ^2^ = 0%, *p* = 0.908) between the two groups.

**Conclusions:**

This meta-analysis confirmed the beneficial effects of SGLT-2i on reversal of left ventricular remodeling. The LVH regression was more pronounced in studies of type 2 diabetes patients receiving SGLT-2i than placebo.

## Introduction

Diabetes is one of the top 10 causes of death in the world, and it also has a detrimental effect on the cardiovascular system, such as peripheral artery disease, left ventricular hypertrophy (LVH), coronary heart disease, arrhythmias, and sudden death (1, 2). Among these complications, LVH is a common disease in type 2 diabetes patients and has been considered as an independent predictor of cardiovascular disease in clinical practice. One of the main causes of LVH is that the heart overworks due to high blood pressure, and cardiomyocytes overcompensate. LVH could also be caused by obesity, and the effects of obesity on the left ventricular structure and function have been described as determining eccentric LVH and diastolic dysfunction (3, 4). Briefly, type 2 diabetes and its associated comorbidities, such as hypertension and obesity, could induce an increase in the LVH as a mechanism of adaptation to a chronic inflammatory state, resulting in an eccentric ventricular remodeling and impairment in myocardial function (5). Thus, new types of blood glucose-lowering agents, which can improve glycemic control along with reducing body weight and controlling blood pressure, are required for diabetes therapy.

Although there are several types of diabetes drugs available in the clinic, evidence of cardiovascular benefit is insufficient. Sodium-glucose co-transporter 2-inhibitors (SGLT-2i) are the first class of hypoglycemic drugs that have been recently shown to be able to reduce the risks of heart failure hospitalization and cardiovascular death (6). By inhibiting SGLT-2 receptor in the kidneys, SGLT-2i can increase urinary glucose excretion, decrease the renal threshold for glucose excretion, and reduce sodium reabsorption, thus reducing the plasma glucose levels, decreasing osmotic diuresis and inducing urinary caloric loss. The urinary caloric loss and osmotic diuresis help to reduce body weight and blood pressure, thereby achieving a cardiovascular benefit (7). However, there is a lack of sufficient evidence of the SGLT-2i effect on cardiac ventricular hypertrophy and function. Therefore, this meta-analysis was undertaken to evaluate the effect of SGLT-2i on cardiac structure and function in patients with type 2 diabetes.

## Materials and methods

### Data sources

The databases including PubMed, Embase, Medicine, Cochrane, and Web of Science were systematically searched for literatures published before August 2022 using the following keywords: “SGLT-2i”, “ventricular hypertrophy”, “dapagliflozin”, “canagliflozin”, “empagliflozin”, “ipragliflozin”, “luseogliflozin”, “tofogliflozin”, “heart failure”, and “cardiac outcome”. All identified articles were manually searched.

### Study selection

**Figure 1:**
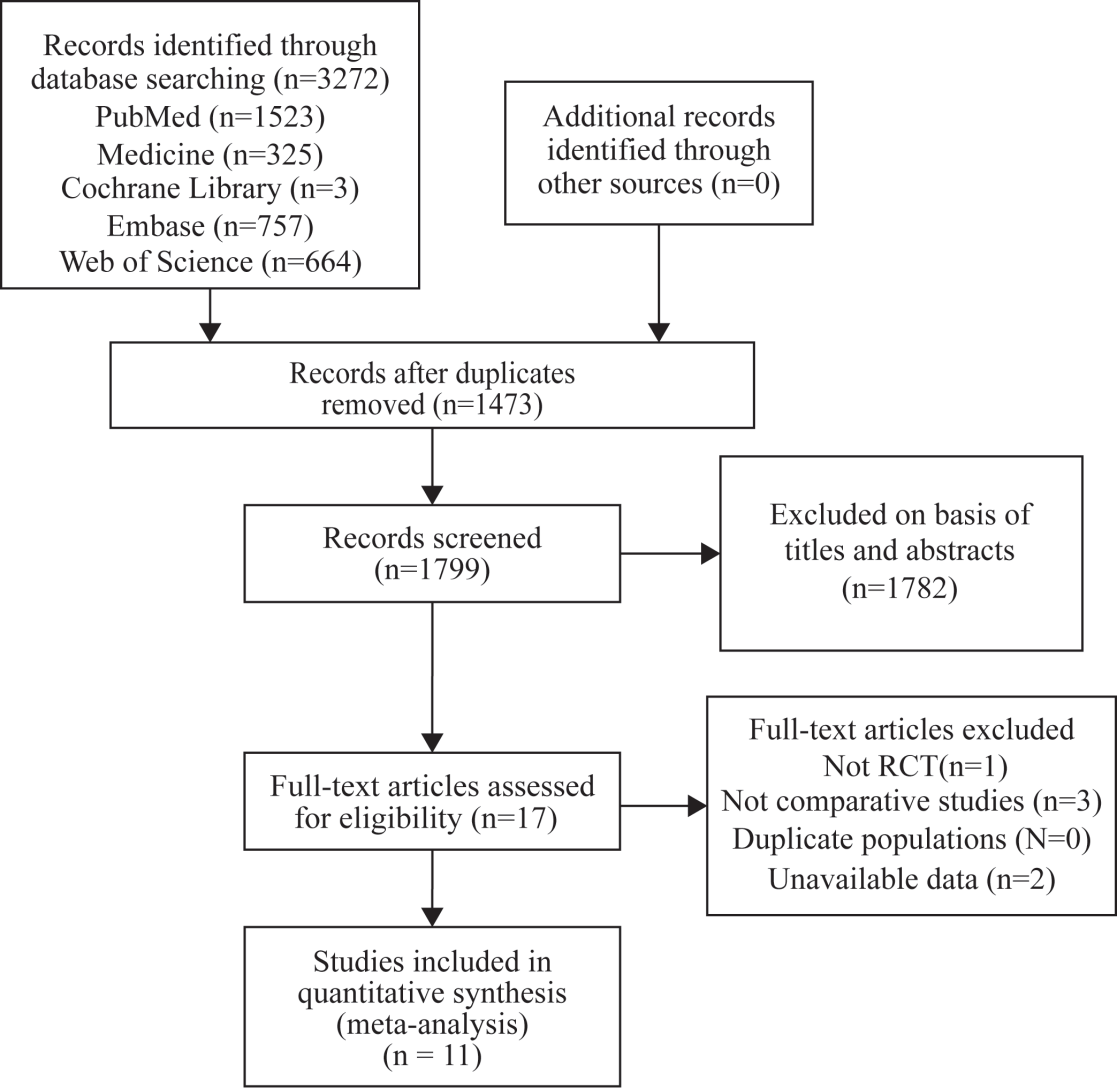
Identification of eligible articles.

We screened articles according to the following criteria: (1) All selected studies must be randomized controlled trials (RCTs) with an experimental group using SGLT-2i and a placebo group using other hypoglycemic drugs or placebo. (2) All patients enrolled in the study must have type 2 diabetes. (3) The outcomes should be ventricular hypertrophy relevant indicators. (4) Each study duration should be at least 12 weeks. The screening process has been shown in the following diagram (**Figure 1**).

### Data extraction

This meta-analysis collected 11 articles of RCTs. The data were extracted in the following format: the first author, published time, simple size, gender, age, research duration, body mass index (BMI), comparison, cardiac imaging method, left ventricular mass (LVM), left ventricular mass index (LVMI), left ventricular end-diastolic volume (LVEDV), left ventricular end-systolic volume (LVESV), left ventricular ejection fraction (LVEF), and ratio of early mitral inflow velocity to atrial inflow velocity (E/A). If there were any disagreements between the two investigators, the decision will be made by a third investigator.

### Statistical analysis and quality assessment

Drafted on the basis of a preset protocol registered with PROSPERO 2022 (CRD42022342371), the present meta-analysis was conducted in line with the preferred reporting items for systematic reviews and meta-analyses (PRISMA) statement (8). Stata 16.0 was used in the present meta-analysis. The standard mean difference (SMD) with 95% confidence intervals (CIs) was used to estimate the pooled effects for continuous variables. The *I*² statistic was used for assessing heterogeneity with a value < 25% indicating low heterogeneity, a value between 26% and 50% indicating moderate heterogeneity, and a value > 50% indicating high heterogeneity. If *I*² < 50%, a fixed-effect model was used; otherwise, a random-effect model was adopted. Sensitivity analyses were used to identify potential sources of the between-study heterogeneity if necessary. Subgroup analyses were conducted by cardiac imaging method [magnetic resonance imaging (MRI) or echocardiography (ECHO)]. The risks of bias assessment of RCTs were carried out with Cochrane’s Risk of Bias 2 (RoB2) tool. The RoB2 tool assessed quality on 5 domains, including the randomization process, deviations from intended interventions, missing outcome data, measurements of the outcome and selection of the reported result. *p* < 0.05 was regarded as statistically significant.

## Results

### Description of studies

**Figure 2:**
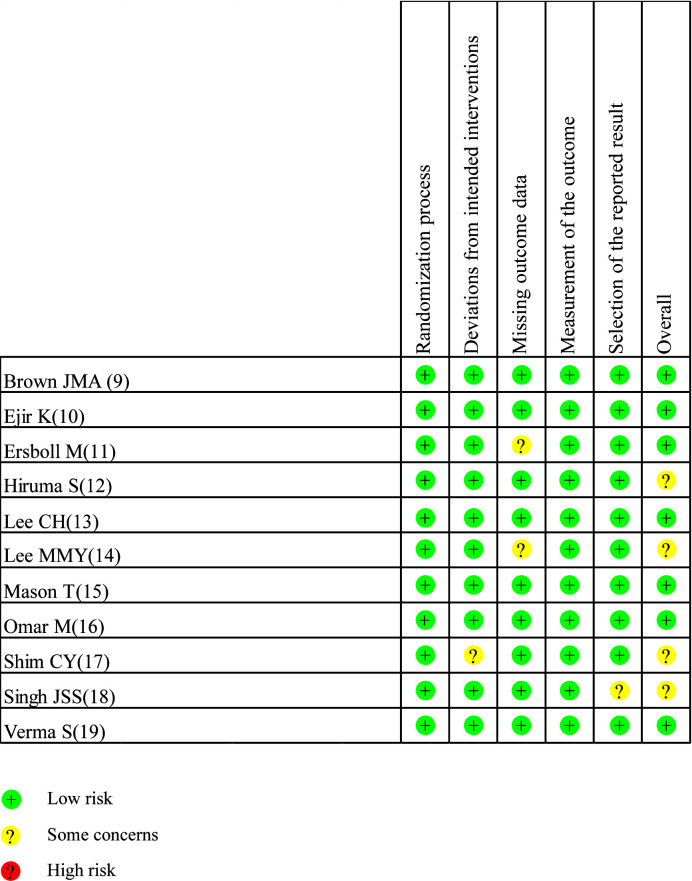
Risk of bias assessment for RCTs.

A total of 3,272 articles were found by our search method, of which 17 underwent full-text review. After 6 inappropriate articles were excluded, 11 articles were included in the meta-analysis. All the characteristics of the included studies are shown in **Table 1**. **Table 2** shows the main results of the included studies. A total of 984 patients were included in the study. Results of the quality assessment of all included studies were satisfying and are shown in **Figure 2**.

**Table 1:** Characteristics of the 11 included studies.

Study	Year	Country	Sample size	Female	Age(year)	Baseline BMI(Kg/m^2^)	Comparison	Cardiac Imaging method	Research duration (month)
Brown JMA (9)	2020	UK	32/34	12/16	64.25/66.74	32.3/32.59	Dapagliflozin 10 mg/day placebo	MRI	12
Ejiri K (10)	2020	JPN	83/82	28/34	71.7/74.6	25.4/25.3	luseogliflozin 2.5 mg/day Voglibose 0.2 mg/day	ECHO	3
Ersboll M (11)	2022	DK	45/42	11/6	66.2/66.7	31.2/29.1	Empagliflozin 10 mg/day placebo	ECHO	3
Hiruma S (12)	2021	JPN	22/22	5/6	52.8/47.8	28.6/30.0	Empagliflozin 10 mg/day Sitagliptin 50 mg/day	ECHO	3
Lee CH (13)	2022	China HK	30/30	14/10	56.9/60.6	26.4/26.9	Dapagliflozin 10 mg/day Sitagliptin 100 mg/day	ECHO	6
Lee MMY (14)	2020	UK	42/50	18/10	68.2/69.2	30.9/30.4	Empagliflozin 10 mg/day placebo	MRI	6
Mason T (15)	2021	CAN	39/35	5/1	62/64	28/26	Empagliflozin 10 mg/day placebo	MRI	6
Omar M (16)	2021	DK	95/95	16/12	65/63	29/29	Empagliflozin 10 mg/day placebo	ECHO	3
Shim CY (17)	2020	USA	30/30	NR	NR	NR	Dapagliflozin 10 mg/day placebo	ECHO	6
Singh JSS (18) {Brown, 2020 #12}	2020	UK	28/28	10/9	66.9/67.4	33/32	Dapagliflozin 10 mg/day placebo	MRI	12
Verma S (19)	2019	CAN	44/46	5/2	64/64	26.7/26.6	Empagliflozin 10 mg/day placebo	MRI	6

Values are all given as SGLT-2i/placebo group; NR:not report; BMI, Body Mass Index; UK, United Kingdom; JPN, Japan; CAN, Canada; USA, the United States of America; HK, Hong Kong: DK, Denmark; MRI, magnetic resonance imaging; ECHO, echocardiography.

**Table 2:** The outcome changes of 11 included studies.

Study	LVM	LVMI	LVEF	LVEDV	LVESV	E/A
Brown JMA (9)	-3.95/-1.13	-0.58/-0.38	1.45/0.66	NR	NR	NR
Ejiri K (10)	NR	-4.23/2.29	2.78/2.95	NR	NR	3.42/6.95
Ersboll M (11)	NR	-11.5/-1.4	-0.9/-1.6	-5/-4.2	-1/-0.8	0/0
Hiruma S (12)	-1.5/-6.0	-1.5/-6.0	0.1/-1.6	NR	NR	0.04/0.00
Lee CH (13)	–1.03/0.27	NR	0.33/-0.15	NR	NR	–0.02/-0.03
Lee MMY (14)	-5.1/-2.5	-2.7/-1.3	1.8 /1.2	-17.3/0.6	-15.1/-2.8	NR
Mason T (15)	NR	-2.7/-0.1	1.3/-1.0	NR	NR	NR
Omar M (16)	NR	-3.7/5.1	2.4/1.0	-8.9/2.8	-8.5/0.2	NR
Shim CY (17)	NR	-2.5/0.2	NR	NR	NR	NR
Singh JSS (18) {Brown, 2020 #12}	NR	4.0/0.6	2.6/1.4	-7.7/-24	-8.9/-18.8	NR
Verma S (19)	-4.7/-0.39	-2.6/-0.01	0.72/1.0	-2.9/-4.3	-1.9/0.3	NR

Values are all given as SGLT-2i/placebo group; NR, not report; LVM, left ventricular mass; LVMI, left ventricular mass index; LVEF, left ventricular ejection fraction; LVEDV, left ventricular end-diastolic Volume; LVESV, left ventricular end-systolic volume; E/A, the ratio of early mitral inflow velocity to atrial inflow velocity.

### Efficacy

#### LVM

**Figure 3:**
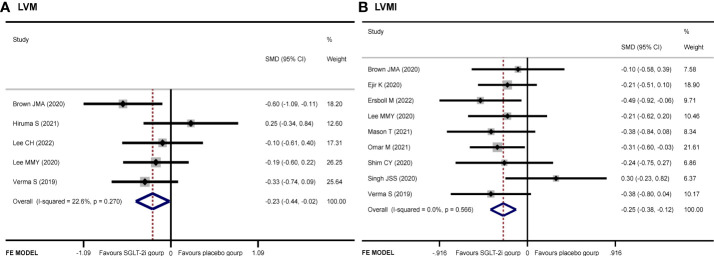
Forest plot of **(A)** LVM and **(B)** LVMI.

**Figure 4:**
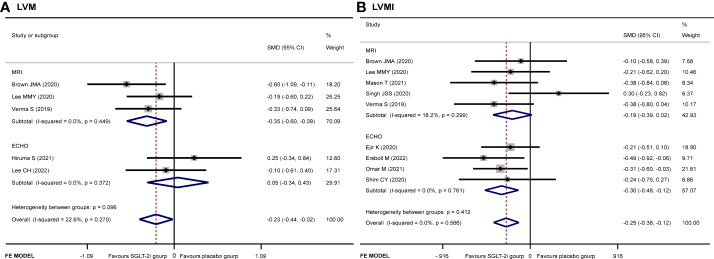
Subgroup analysis of **(A)** LVM and **(B)** LVMI.

Five articles (9, 12–14, 19) recorded the changes of LVM. The SGLT-2i group had a significant reduction in LVM compared with the placebo group (SMD −0.23, 95% CI −0.44 to −0.02, *I* ^2^ = 22.6%, *p* = 0.034) (**Figure 3A**). No publication bias by either Begg’s (*p* = 0.462) or Egger’s (*p* = 0.412) test was identified. The subgroup of MRI demonstrated that patients receiving SGLT-2i had a better LVM regression than the placebo group (SMD −0.35, 95% CI −0.60 to 0.09, *I* ^2^ = 0.0%, *p* = 0.007). Meanwhile, subgroup analysis showed that no statistically significant result was obtained for the ECHO group (**Figure 4A**).

#### LVMI

A total of 931 patients included in nine articles (9–11, 14–19) documented the changes in LVMI data. The SGLT-2i group had a better effective LVMI reduction than the placebo group (SMD −0.25, 95% CI −0.38 to −0.12, *I* ^2^ = 0.0%, *p* = 0.000) (**Figure 3B**). No publication bias was found by either Begg’s (*p* = 0.466) or Egger’s (*p* = 0.390) test. We performed a subgroup analysis for LVMI. The results showed that no statistically significant result was obtained in the studies using MRI, while the results of the ECHO group showed that SGLT-2i had a significant reduction in LVMI (SMD −0.30, 95% CI −0.48 to −0.12, *I* ^2^ = 0.0%, *p* = 0.001) (**Figure 4B**).

#### LVEF

**Figure 5:**
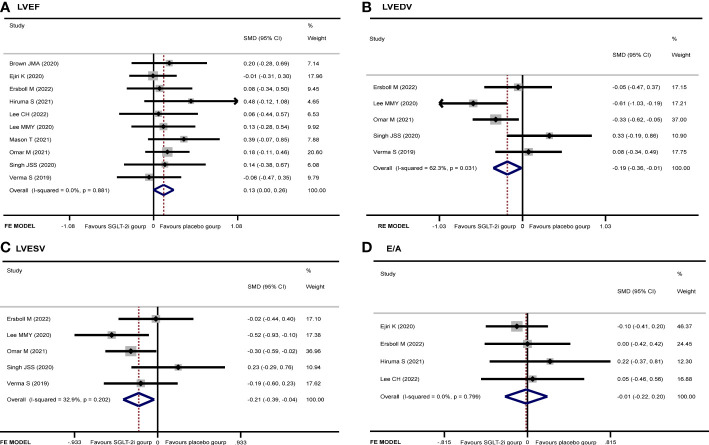
Forest plot of **(A)** LVEF, **(B)** LVEDV, **(C)** LVESV, and **(D)** E/A.

After extracting data from 10 studies (9–16, 18, 19), the pooled result showed that there was no significant difference in LVEF (SMD 0.13, 95% CI 0.00 to 0.26, *I* ^2^ = 0.0%, *p* = 0.050) between the two groups (**Figure 5A**). The subgroup analysis according to the cardiac imaging method demonstrated that no significant difference in LVEF between the two groups was recorded (**Supplementary Figure 1**). Begg’s (*p* = 0.283) and Egger’s (*p* = 0.227) tests both discovered no publication bias.

#### LVEDV

**Figure 6:**
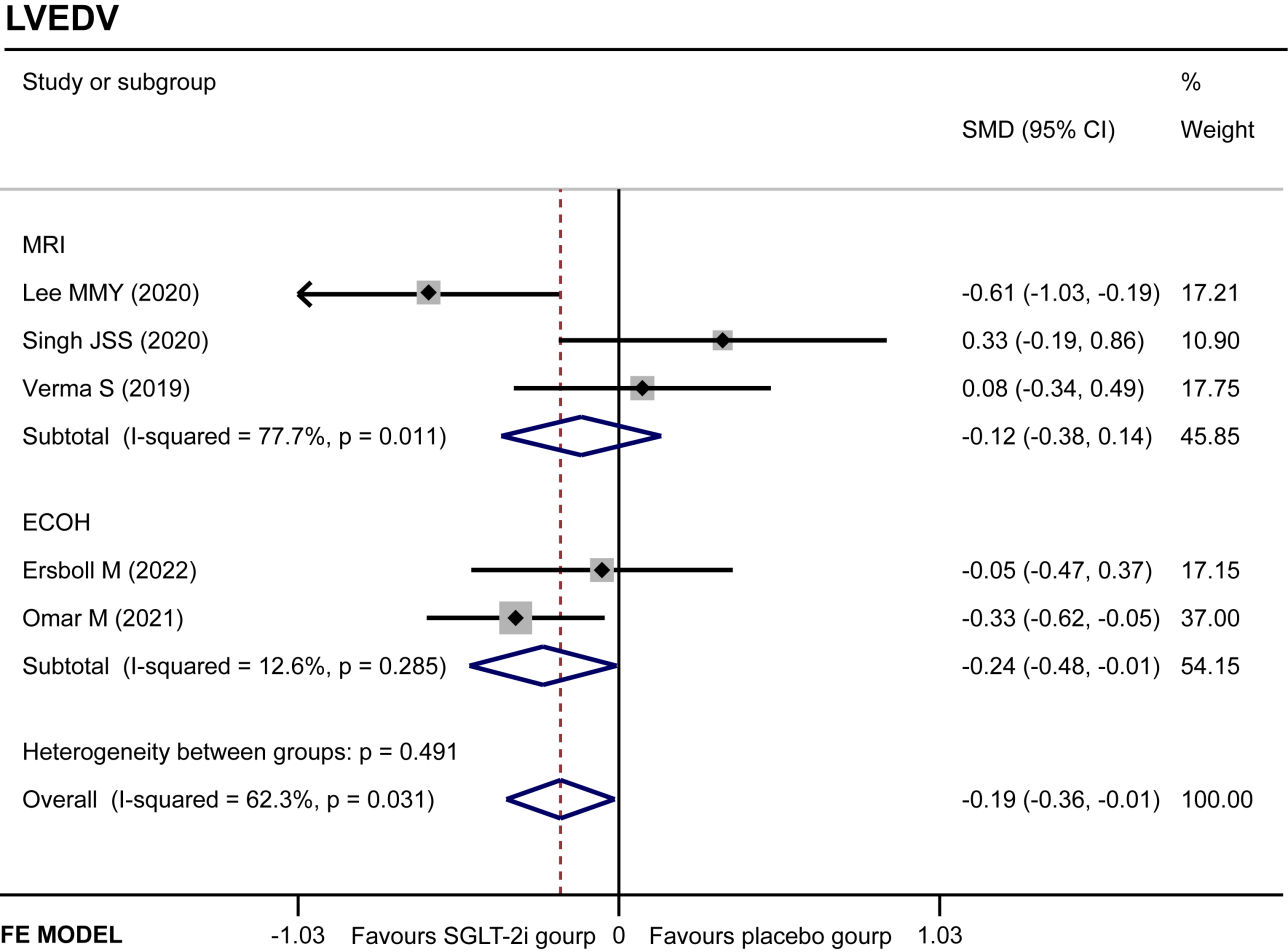
Subgroup analysis of LVEDV.

Five studies (11, 14, 16, 18, 19) regarded the LVEDV. A random-effect model revealed that the SGLT-2i group had a better effect in LVEDV reduction than the placebo group (SMD −0.19, 95% CI −0.36 to −0.01, *I* ^2^ = 62.3%, *p* = 0.035) (**Figure 5B**). None of the studies were found to have a significant influence on the result in a sensitivity analysis (**Supplementary Figure 2**). The subgroup analysis according to the ECHO group showed that SGLT-2i had a better reduction in LVEDV compared with the placebo group (SMD −0.24, 95% CI −0.48 to −0.01, *I* ^2^ = 12.6%, *p* = 0.043) (**Figure 6**). Neither Begg’s (*p* = 0.462) test nor Egger’s (*p* = 0.325) test showed publication bias.

#### LVESV

There were 5 studies (11, 14, 16, 18, 19) that discussed the LVESV; the pooled result indicated that the SGLT-2i group had a better reduction in LVESV compared with the placebo group (SMD −0.21, 95% CI −0.39 to −0.04, *I* ^2^ = 32.9%, *p* = 0.017) (**Figure 5C**). The subgroup analysis of the cardiac imaging method demonstrated that no significant difference in LVESV between the two groups was recorded (**Supplementary Figure 3**). No publication bias was found by either Begg’s (*p* = 0.221) or Egger’s (*p* = 0.324) test.

#### E/A

A total of four articles (10–13) were included in the E/A study. There was no significant difference in E/A between the experimental and control groups (SMD −0.01, 95% CI −0.22 to 0.20, *I* ^2^ = 0%, *p* = 0.908) (**Figure 5D**). Neither Begg’s (*p* = 0.089) test nor Egger’s (*p* = 0.024) test showed publication bias.

## Discussion

The effect of SGLT-2i on LVH was shown to be relative satisfaction in the present study. Although there were no notable differences in the changes of LVEF and E/A between these two groups, the improvement effects on LVM, LVMI, LVEDV, and LVESV in the SGLT-2i group were better than those in the placebo group. Moreover, the subgroup of MRI raised the possibility that SGLT-2i might promote LVM regression, and the subgroup of ECHO showed that the SGLT-2i group had significant reductions in LVMI and LVEDV.

It is well known that LVM is a significant indicator for evaluating LVH. Several factors could explain the improvement effect of LVM in the SGLT-2i group. First, a study demonstrated that SGLT-2i have a beneficial effect on decreasing blood volume, which induces decrease of blood pressure in diabetic patients. Great burden caused by hypertension may lead to an increase in the size of cardiomyocytes, and the heart develops hypertrophy to adapt to the changes in the cardiovascular system (20). Therefore, SGLT-2i can indirectly reduce LVM by lowering blood pressure. However, some small reduction in the control group of LVM may account for the fact that all participants were closely monitored by visits (9). Second, there is another mechanism to explain the effect of SGLT-2i on LVH. In chronic inflammatory disorders (especially those leading to heart failure with preserved ejection fraction), the epicardium becomes a site of deranged adipogenesis, leading to the secretion of proinflammatory adipokines that can cause atrial and ventricular fibrosis (21). To address this issue, SGLT-2i, such as luseoglifozin, ipraglifozin, and canaglifozin, are used to reduce epicardial fat accumulation (22–24). Third, SGLT-2i directly cause body weight loss *via* glucose excretion (calorie loss) in the kidneys (25). Accordingly, multiple studies have shown improvements in LVM after weight loss procedures (26). Taken together, it can be known that SGLT-2i could reduce LVM by reducing BP, epicardial adipose tissue (EAT), and body weight.

Among them, LVMI, the main LVH indicator (27), was significantly reduced by SGLT-2i in our study. The diagnosis criteria for LVH using LVMI are ≥ 115 g/m^2^ in male patients and ≥ 95 g/m^2^ in female patients (28). The relationship between LVM and LVMI is as follows: LVMI (g/m²) = LVM/BSA (body surface area). Our analysis found that SGLT-2i had a strong correlation with the reduction in LVM. Therefore, SGLT-2i reduce LVMI mainly through the possible LVM reduction described above. Furthermore, SGLT-2i can reduce body weight, while overweight is a potential risk factor of LVH (29, 30). SGLT-2i may indirectly affect BSA through the effect of weight loss and LVMI reduction. Differently, Singh JSS’s study showed that LVMI was significantly increased in the SGLT-2i group and indicated that other mechanisms should be considered, beyond the established paradigm of left ventricular remodeling, to explain these effects of SGLT2i (18), which also provides a new idea for future research on ventricular hypertrophy. Therefore, further studies focusing on the mechanisms of the effects of SGLT2i on LVMI are still needed.

LVEDV and LVESV are commonly used to calculate the LVEF to reflect the ejection function of the heart. An increase in LVEDV and an increase in LVESV are considered as two harmful factors to the heart (14). In our meta-analysis, we obtained the results that SGLT-2i could reduce LVEDV and LVESV, which were consistent with some studies’ conclusions (31). The possible reason is that a slight diuretic effect of SGLT-2i can promote urination and reduce pre-load, and the consequent reduction in left ventricular stretch may lead to a reduction in LVEDV and LVESV (14, 32). More evidence and research are needed in the future research to prove this hypothesis.

LVEF as a common metric of cardiac performance in clinical practice, its value is generally thought to be above 50%. The pooled result of our study revealed that there were no significant differences in LVEF between the two groups. However, this does not mean that SGLT-2i cannot improve left ventricular function at all. According to the results of the current studies, the effect of SGLT-2i on increasing LVEF was well established (33). Several factors might explain their findings. Some opinions consider that both heart rate and fiber shortening affect ejection fraction during the measurement process, but it is more influenced by LVEDV. This could also be explained by the reduction in left ventricular volume impacted by SGLT-2i (34). In addition, some patients with obesity might present mild to moderate left systolic dysfunction, which can be proven by a decreased ejection fraction. Importantly, SGLT2i can improve ejection fraction with the weight loss (35, 36). However, more research is needed to testify this.

Another concern is E/A, which is one of the left ventricular function measurements in the cardiac color ultrasound report and can be used to evaluate the diastolic function of the ventricle. The normal value is E/A > 1, and E/A < 1 is the manifestation of reduced ventricular diastolic function. In the present study, no statistically significant difference between the two groups was observed in the included studies (10–13). In contrast, some researchers believe that SGLT-2i could increase E/A after taking SGLT-2i. Interestingly, a recent research found that SGLT-2i can reduce left ventricular mass and attenuate cardiomyocyte hypertrophy in conjunction with diminished wall stress and improve diastolic function in a mouse research model (37). In order to get more convincing results, it is suggested to include more data for analysis.

In addition, the cardiac imaging methods of identified studies were different, including MRI and ECHO. To our knowledge, although ECHO is frequently used in clinical practice and detects all but the mildest degrees of LVH, ECHO has substantial measurement variability and remarkable rates of non-evaluability (38). Cardiac MRI provides three-dimensional coverage of the left ventricle without image quality limitations related to ultrasound transmission, resulting in more accurate in estimating dimensions of the left ventricle than ECHO (39, 40). In the present study, the results of the subgroup analysis of the two cardiac imaging methods remain controversial and require further study.

## Limitations

There were several limitations in our meta-analysis. First, we only collected 11 studies and the included samples were mostly from Japan, USA, UK, and Canada. These samples are not universal. Second, the sample size of the test groups was small. Third, the drugs used in placebo groups may have an unknown effect on the cardiovascular system, which may impact the outcome. In addition, due to the short experimental time of our included studies, the long-term effects of the SGLT-2i might be ignored.

## Conclusions

In summary, SGLT-2i have beneficial effects on LVH in type 2 diabetes patients. SGLT-2i can significantly reduce LVM and LVMI, which are important indicators of ventricular hypertrophy, indicating that SGLT-2i play a certain role in the treatment of LVH. Large sample sizes and long-term follow-up studies are still needed to further identify differences.

## Author contributions

YW and XH originated and designed the study. YZ, ZZ, and QZ conducted the literature search, data extraction, data analysis, and interpretation and drafting of the manuscript. SY conducted the data interpretation and methodology. YW and BC conducted critical revision of the article and final approval. All authors contributed to the article and approved the submitted version.

## Supplementary material

Supplementary Figure 1 The sensitivity analysis of LVEDV.

Supplementary Figure 2 Subgroup analysis of LVEF.

Supplementary Figure 3 Subgroup analysis of LVESV.
