# Supplementary figures and images for: Effect of sodium-glucose cotransporter protein-2 inhibitors on left ventricular hypertrophy in patients with type 2 diabetes: A systematic review and meta-analysis
Source: Front Endocrinol (Lausanne). 2023 Jan 9;13:1088820. doi: 10.3389/fendo.2022.1088820 (PMC9868415; doi:10.3389/fendo.2022.1088820)

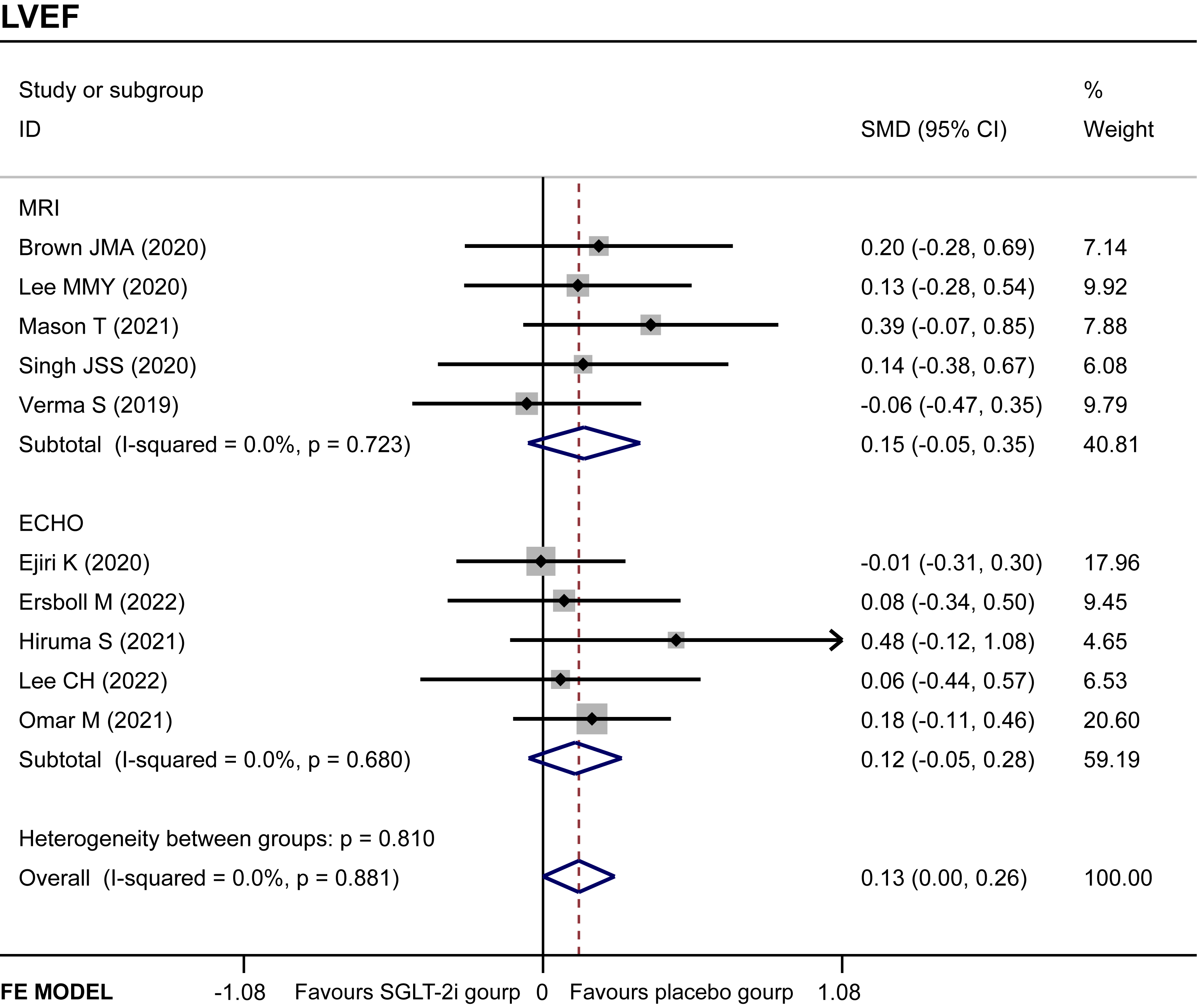

Supplement: Supplementary Figure 1 — The sensitivity analysis of LVEDV. [file Image_1.tif]

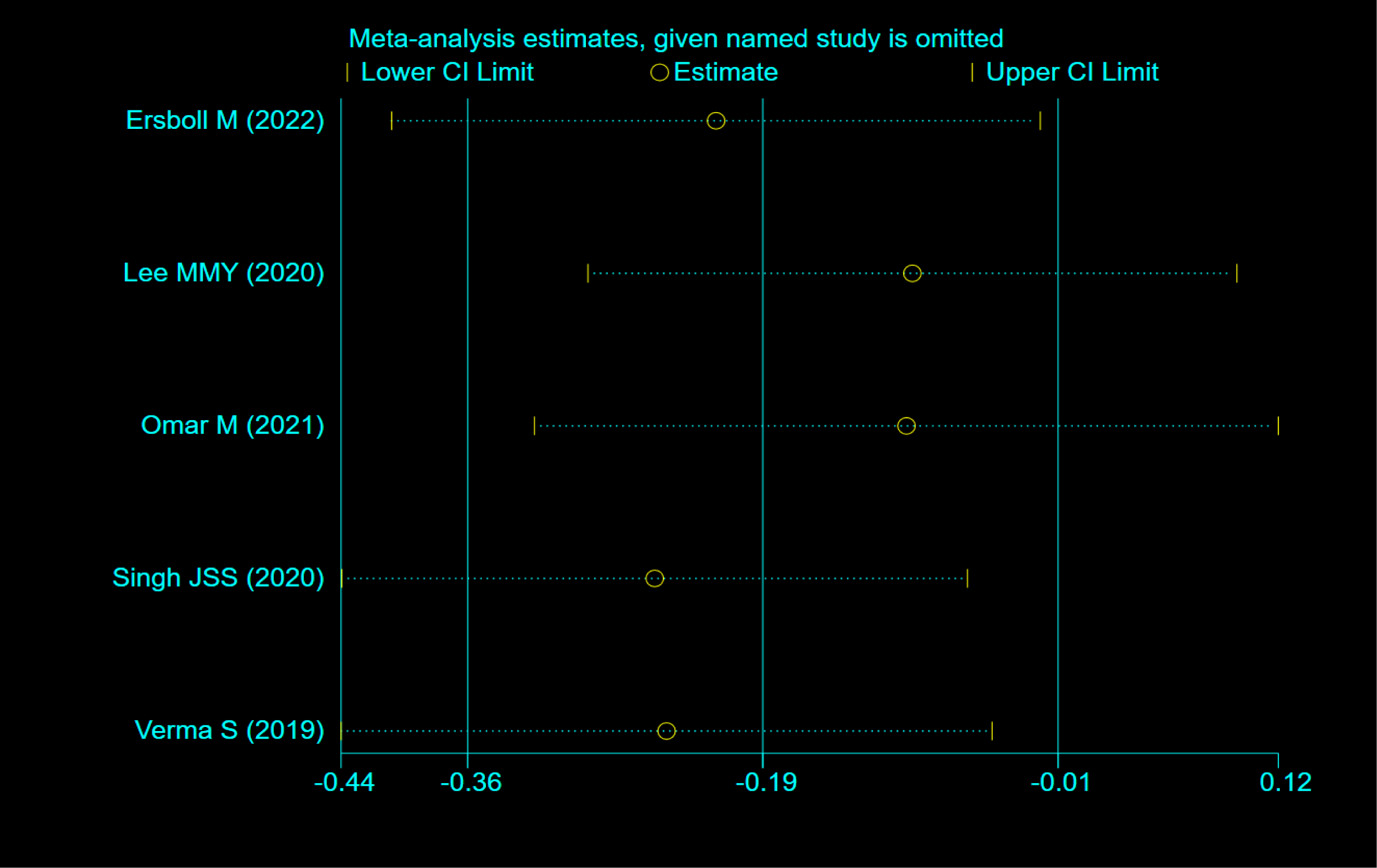

Supplement: Supplementary Figure 2 — Subgroup analysis of LVEF. [file Image_2.tif]

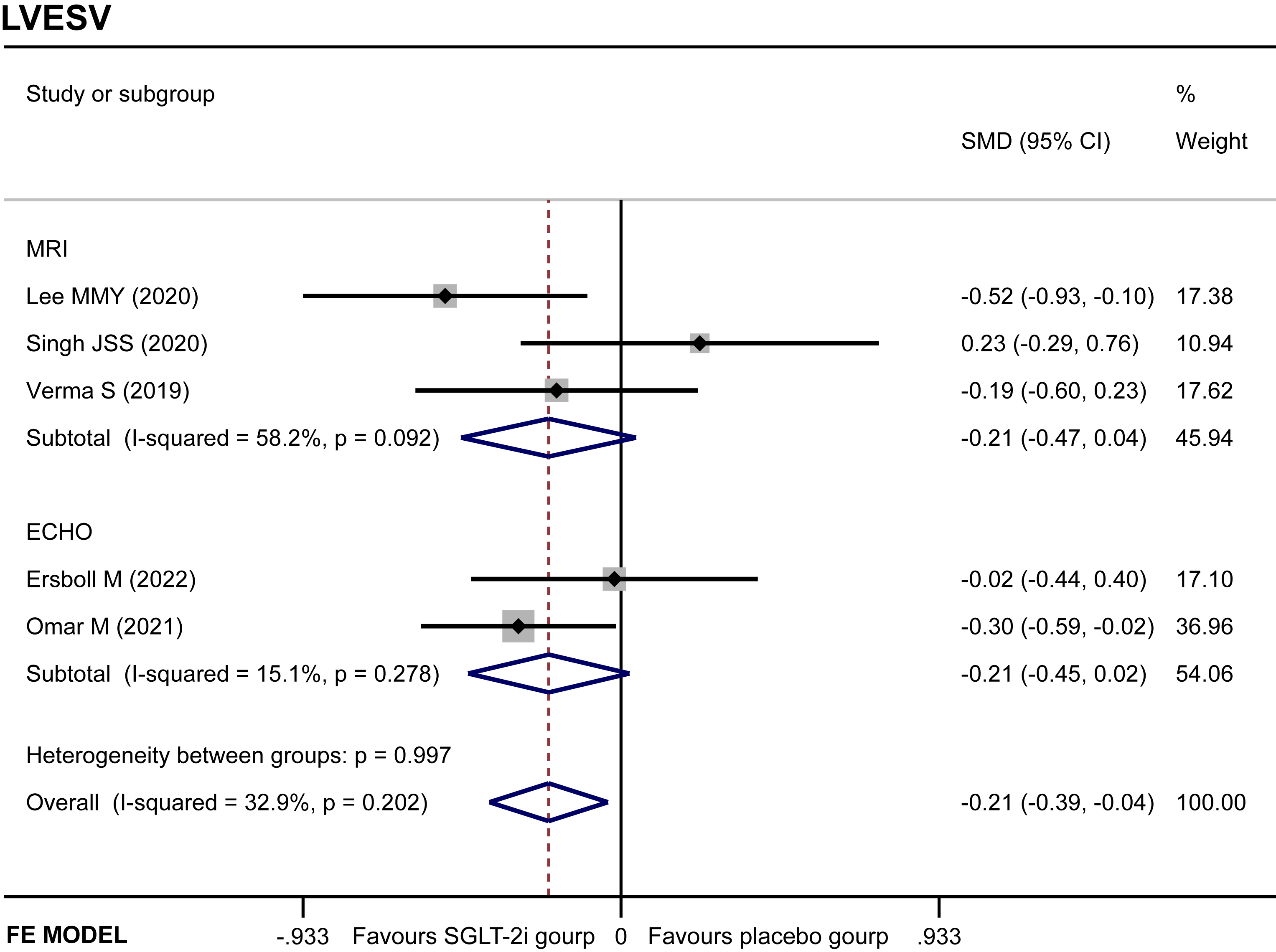

Supplement: Supplementary Figure 3 — Subgroup analysis of LVESV. [file Image_3.tif]
